# 

**Published:** 1890-06-28

**Authors:** 


### NOTICE TO CORRESPONDENTS.

All MS., Letters, Books for Review, and other matters intended for the Editor should be addressed The Editor, The Lodge, Porchester Square, London, W.

The Editor cannot undertake to return rejected M.S., even when accompanied by stamped directed envelope.

**Notice to Advertisers and Subscribers.**

All Advertisements, Orders for Copies of The Hospital, and Business Communications should be addressed to The Publisher, *not to the Editor*, The Hospital, 140, Strand, W.C.

**Bound Volumes of "The Hospital."**

Vol. VI., containing full page portraits of T.R.H. the Prince and Princess of Wales, and Miss Florence Nightingale, is obtainable at 4s., post free.

Orders will be received for Vol. VII., 4s. post free. Cases for binding may be had of the Publisher, at 1s. 6d. each.

**To Residents, Secretaries, and Matrons.**

The Editor will be glad to have the Regulations and each Report as issued by Asylums, Hospitals, Convalescent and Nursing Institutions, as well as those of other Charities, and an early copy in duplicate of all Appeals and Festival Notices, etc., addressed to *The Editor, The Lodge, Porchester Square, London, W.* Any superintendent, secretary, matron, or other chief official who regularly sends such information may be registered, on application to the Publisher, to receive free of cost a cover for binding The Hospital in half-yearly volumes.

BURDETT'S HOSPITAL ANNUAL FOR 1890

Is now ready, and may be obtained of the Publisher, The Hospital Offices, 140, Strand, W.C., price 2s. 6d. limp boards, or 3s. 6d. cloth. All orders must be accompanied by a remittance.

### Scraps and Gleanings.

The Smedley Memorial Hospital at Matlock is very full just now.

Stockwell Orphanage held its Festival on June 19th, Mr. Spurgeon's birthday.

There was a garden party at the Cancer Hospital, at Brompton, on June 27th.

The question of an infectious hospital for Birstall is again before the Local Board.

Hospital Sunday at Belfast resulted in a collection of £324, about £60 more than last year.

An interesting account of "Hypnotism in Paris Hospitals" appeared in the *Tablet* for June 14th.

The Sevenoaks Vine Hip Hospital needs wider support; 87 of its patients have been from London.

Dr. John Marshall is to receive the honorary degree of Doctor of Medicine from Dublin University.

At Grange-over-Sands the Convalescent Home for Friendly Societies members is being well appreciated.

Lady Hart Dyke opened a very successful bazaar, lately held at Sidcup, in aid of the new Cottage Hospital.

Deighton lately held an infirmary demonstration; a procession, sports, dancing and fireworks made up a happy day.

The *Liverpool Mercury* is giving a series of interesting articles, dealing so far with Hospitals, entitled "Our Charities."

Blackpool has now got such a handsome infectious hospital, the Corporation can't help a guilty longing for an epidemic.

Mrs. W. H. Smith had an afternoon "At Home," on June 27th, in the interests of the Children's Country Holidays Fund.

Gloucester Eye Institution reports that it admitted 81 in-patients last year, and the expenditure and income have balanced.

Archbishop Walsh has visited Dublin Royal Hospital for Incurables, and expressed his pleasure at finding the patients so well cared for.

The Medical Congress in Berlin has decided to grant the application for admission to its sittings, presented by an American lady physician.

The Middlesex Hospital Medical Society will give a conversazione in the grounds of the hospital, on July 4th, from nine till twelve o'clock.

General Goldsworthy last week headed a deputation which waited on Mr. Long, M.P., to protest against the enlarging of the Fulham Fever Hospital.

Canon Wilberforce presided last week at a conference of the Society for the Protection of Animals from Vivisection, and delivered a rousing speech.

The London Throat Hospital issues its third annual report, in which it states that 1,262 new patients availed themselves of the benefits of the hospital last year.

The annual meeting of subscribers to Queenstown General Hospital was held on June 16th; 114 patients were received last year, and there were eight deaths.

Walthamstow Dispensary, founded in 1873, treated 1,389 patients last year. The expenditure exceeded the income by £66, and increased subscriptions are asked.

The Duke of Edinburgh is going to open the new Victoria Hospital at Folkestone, and the Mayor and Town Council mean to give His Royal Highness a grand reception.

The small-pox epidemic has broken out in several villages in Egypt. The Government has granted a sum of £2,000 to the sanitary department for vaccinating purposes.

A Dublin lady has set up a house for destitute cats, and takes in boarders of the feline race when their own families leave town for a stay by the sea or a run on the Continent.

The St. George's and Westminster Committee of the Hospital Saturday Fund gave their annual concert on June 17th. Mr. Goschen, jun., stated that the collection in their district had risen from £200 to £600.

The French Government has deputed Dr. Paul Thiéry to visit Scotland, Sweden and Norway, Denmark, and Russia, for the purpose of studying the organisation of surgical teaching and of seamen's hospitals.

The new Liverpool Infirmary is rapidly approaching completion, and is expected to be opened a few weeks hence. No final arrangement as to the inaugural ceremony has yet been made, but it is hoped that this will be performed by a representative of the Royal Family.

The inhabitants of West Malling and neighbourhood have shown their appreciation of the services rendered by Dr. Pope, who is about to retire and leave the district, after nearly half a century spent in the active duties of his profession there, by presenting him with a cheque for 100 guineas and an illuminated address.

Having observed the good work being done at the Animals' Institute in alleviating the sufferings of animals, a gentleman has made a handsome offer, if others will come forward to help in forming a similar establishment in the East-end. Particulars can be had from the medical superintendent of the Animals' Institute, 9, Kinnerton Street, Wilton Place.

There was a large gathering at Charing Cross Hospital Medical School last week, to witness the distribution of prizes and certificates to the successful students in the past two sessions by the Dean, Dr. Mitchell Bruce. An encouraging report was presented by the subdean, Mr. Stanley Boyd, which stated that thirty-two successful candidates for last year's vacancies in the Indian, Home, and Naval medical services received a portion of their training at the institution.

